# Specific ICF training is needed in clinical practice: ICF framework education is not enough

**DOI:** 10.3389/fresc.2024.1351564

**Published:** 2024-07-08

**Authors:** Jaana Paltamaa, Ellen van Lingen, Christine Haumer, Anita Kidritsch, Ingrid Aerts, Laura Mutanen

**Affiliations:** ^1^ The School of Health and Social Studies, Jamk University of Applied Sciences, Jyväskylä, Finland; ^2^ Rehabilitation Centre “Revalidatie Friesland”, Beetsterzwaag, Netherlands; ^3^ Moorheilbad Harbach Gesundheits- & Rehabilitationszentrum, Moorbad Harbach, Austria; ^4^ Institute of Health Sciences, St. Poelten University of Applied Sciences, Vienna, Austria; ^5^ Department of Health and Science, AP University of Applied Sciences and Arts, Antwerp, Belgium; ^6^ Coronaria Rehabilitation and Therapy Services (Coronaria Contextia Ltd), Tampere, Finland

**Keywords:** functioning, ICF, training, education, rehabilitation, implementation, interprofessional collaboration, person-centered

## Abstract

The use of a common language in interprofessional collaboration is essential. The World Health Organization's International Classification of Functioning, Disability and Health (ICF) has been identified as a unifying framework for interprofessional collaboration and the identification of client needs. Higher education institutions (HEIs) offer ICF framework education to students but is it enough to enable graduated professionals to implement the ICF in clinical work? In our experience, the ICF education provided by HEIs does not meet the requirements of clinical practice, which might be due to gaps in teaching ICF to students (education) and specific requirements for teaching ICF to professionals already working in rehabilitation (training). This paper discusses the need for the ICF training in practice and ways to address it. Although many rehabilitation center professionals had previously received ICF education provided by the HEIs, the rehabilitation centers felt the need to develop their own practical training materials that could be applied to their own environment. Overall, 18 different ICF-based materials were developed during the Erasmus+ project called INPRO to promote person-centered and interprofessional practice in the rehabilitation centers. The practical training using real cases was considered valuable. It could be further developed in cooperation with HEIs and vice versa. It could also be used to teach students, i.e., future colleagues. To deepen and broaden the integration of the different materials based on the ICF, it is important to continue the interactive discussion between HEIs and clinical practice, and between management and its staff.

## Introduction

The number of elderly or people with chronic illness with reduced functioning is growing, and at least one in three people worldwide will need rehabilitation at some point during their illness or disability (1). At the same time as the demand for healthcare is increasing, the primary care workforce is shrinking, and healthcare costs are rising steadily (2).

The need for rehabilitation is enormous (1). One of the most promising solutions is interprofessional collaboration (3), which can reduce hospital or rehabilitation center stays by improving interprofessional and person-centered collaboration between health and social care professionals (hereafter referred to as “professionals”) (4). Although much attention is paid to interprofessional education and collaborative practice (IPECP), there is a gap between the level of competence of future professionals and the level required in rehabilitation practice (3).

The World Health Organization International Classification of Functioning, Disability and Health, more commonly known as the ICF, defines a universal language for health and disability to facilitate communication between professional groups and collaboration in health care (5). According to the World Health Organization's ICF Practical Manual (6, page 11), “*the ICF is relevant to many activities in clinical practice such as the consideration of health and functioning, setting goals, evaluating treatment outcomes, communicating with colleagues or the person involved”*.

The ICF has been used in many different contexts and for many different purposes around the world (6–10). A recent large-scale survey (10) found that clinical practice was one of the main areas in which the ICF was used in different countries. In clinical settings, the ICF was mainly used in the context of rehabilitation and outcome assessment. This highlights the importance of ICF training for rehabilitation professionals.

Education and training in the ICF framework has been provided since its publication in 2001. The background and objectives of the development of the ICF were first described in a book published by the WHO (5). The model and its components are still valid, and the categories have been undergoing regular updates by WHO since 2011 (11). The ICF Practical Manual (6) and the ICF eLearning Tool (12) have been developed to educate the ICF. The ICF has also been incorporated into medical and health education curricula in countries such as Canada (13, 14), South Africa (15, 16) and Australia (17, 18), but the integration of the ICF into medical and healthcare education programs worldwide has been slow (18). Integration of ICF knowledge into practice also seems lagging.

In a Brazilian survey (19) of more than 1,300 professionals with graduate certificate, most of them claimed to be familiar with the ICF, but the majority reported they did not use the ICF. The study found that a quarter of the respondents had never been in contact with the ICF. This may be due to the following reasons: professionals who graduated before the adoption of the ICF (year 2001); professionals who did not seek for continuing education; or even professionals who were seeking professional development but had not been informed about the ICF. The main reasons for non-implementation of the ICF in the clinical practice was related to the extent and complexity of the ICF.

Using the ICF in clinical practice requires basic knowledge about the ICF (6) and clinical ICF-based applications and tools (20–23). It has been found that interprofessional communication improved with the ICF report (20) and the ICF Checklist of Components improved interprofessional practice in hospitals (21). ICF tools developed for use at different stages of the rehabilitation cycle supported a common understanding of functioning (22). The ICF was used in combination with existing goal-setting tools in clinical practice (23).

It has been argued that the ICF and its tools should be incorporated into the established clinical routines to promote its use among healthcare professionals (22). The need for specific practices and tools was discussed in focus groups (rehabilitation center professionals and HEI lecturers) of the Erasmus+ INPRO - Interprofessionalism in action -project (24) but there was no more specific information on what these tools and practices would be. The focus groups assessed what kind of tools they needed to make better use of the ICF in their rehabilitation work in identifying client-centered needs, clinical reasoning and planning more appropriate interventions. The professionals in the rehabilitation centers had received an ICF framework education provided by the HEIs using the WHO material. They felt that the formal ICF education was not sufficient to support ICF implementation in practice. In our experience, more practical material and training is needed and is discussed in this paper. We use the term “education” to refer to formal ICF education and by “training” we mean learning in clinical practice.

## Development of ICF training for clinical practice

In four focus groups (one in Austria, Belgium, Finland and the Netherlands) rehabilitation center professionals and HEI lecturers assessed what ICF-based materials and training are needed to improve ICF implementation (24).

Based on experiences and needs of the focus group participants (24), the aim was to develop ICF-based materials that promote person-centered and interprofessional practice in the rehabilitation centers and that could also be used for teaching the ICF in higher education. The development focused on clinical practice, but it would be useful if the same practices or tools could be used in HEIs to educate students, that is future colleagues.

**Figure 1:**
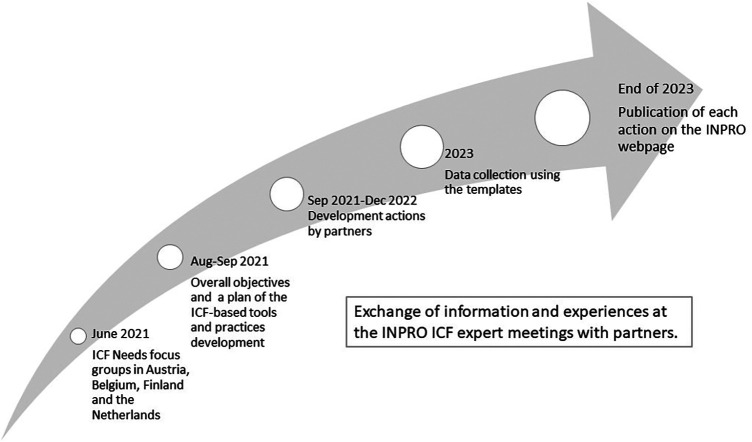
A description of the development process (25).

Between June 2021 and December 2022, a cyclical development process was carried out in collaboration with three rehabilitation centers and four HEIs in Austria, Belgium, Finland, and the Netherlands (25) (Figure 1). The three rehabilitation centers were the cornerstones of the development. The work was guided by an interactive exchange of knowledge, experiences and ICF-based materials and practices, both internationally between partners and nationally between the rehabilitation center and the HEI. The objectives and therefore the actions were individual and varied between partners according to their needs.

**Figure 2:**
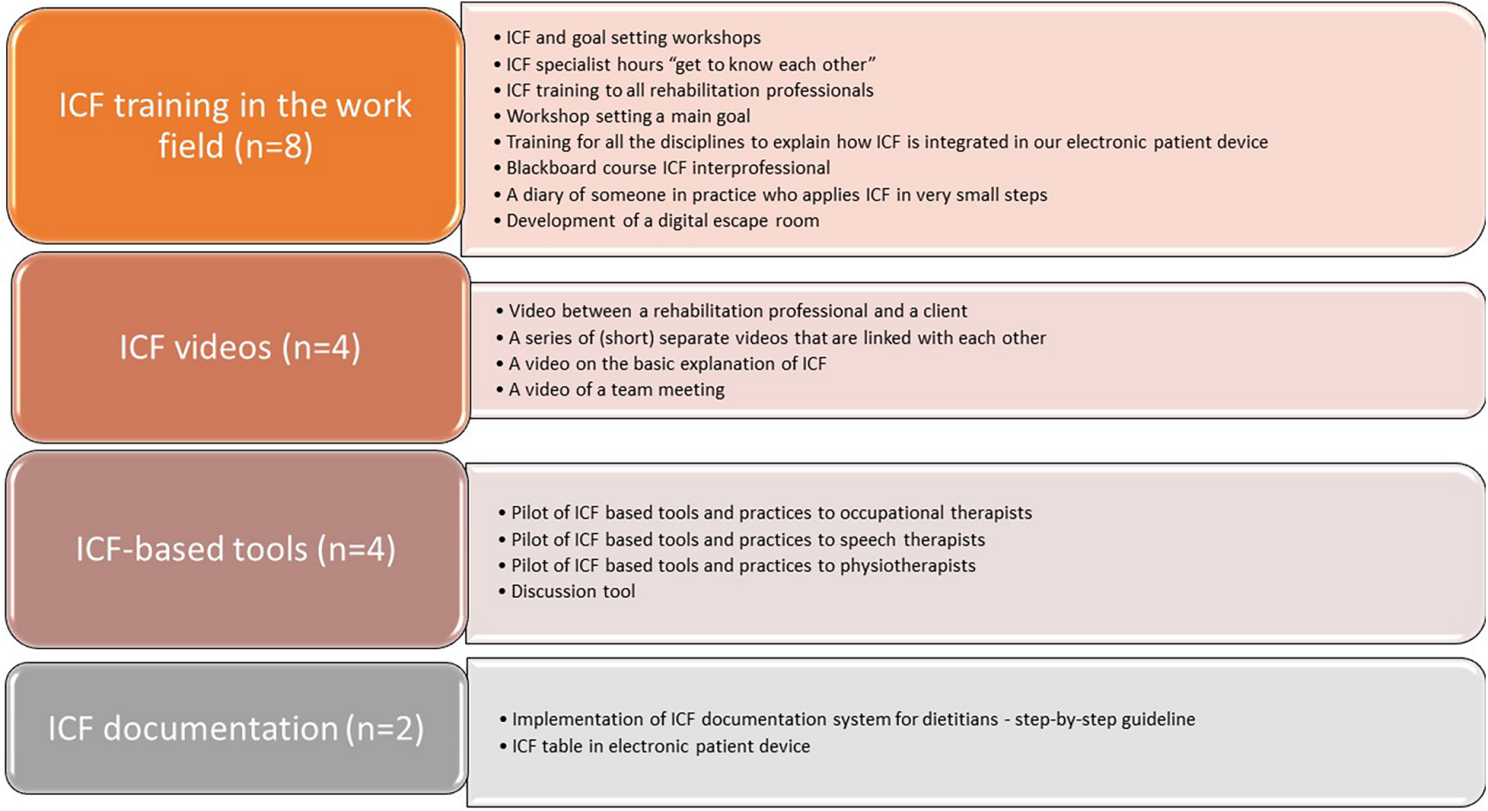
A summary of themes (*n* = 4) and fully implemented ICF-based material and training actions (*n* = 18) (25).

In total, 18 of the 20 planned development actions to support ICF training and use in the workplace were completed (25). Development actions are action steps that rehabilitation centers could implement to support ICF trainings, e.g., a video on the basics of the ICF, and to promote ICF use at the workplace, e.g., pilot of ICF-based tools and practices to professionals of diverse disciplines. Four clear themes emerged: ICF training in the workplace, ICF videos, ICF-based tools and ICF documentation. These development actions are shown in Figure 2. Not all partners developed all the different themes, but each theme was still addressed by one to three different partners.

The greatest need was for dedicated own ICF training (*n* = 8) in rehabilitation centers. This was despite the fact that many of the professionals in the rehabilitation centers had previously received ICF education provided by the HEI. Each HEI offered ICF education in almost all social and health care education programs. This ICF education had mainly reached younger, recent graduated professionals, but this did not seem to be the only reason for the inadequacy of the ICF education in HEIs. In particular, the depth of the content of ICF education varied, i.e., whether only the ICF was taught as a framework or whether codes and qualifiers were also included in the course.

The rehabilitation centers felt the need to develop their own training manual, which contained practical material that could be applied in their own working environment. An important step in developing the use of the ICF in the workplace was therefore to train rehabilitation professionals in the use of the ICF. Part of the material consists of workshops on goal setting, which are accessible to all rehabilitation professionals, including physiotherapists, activity therapists, social workers, psychologists, occupational therapists, music therapists, speech therapists and social workers. In addition, “ICF specialist hours to get to know each other” were developed as well as a material to help the interns make the translation from ICF in education to ICF use in practice. Specific ICF-based tools (*n* = 4) and videos (*n* = 4) were developed to support ICF training and use in clinical practice. ICF-based tools, such as the Rehabilitation Problem Solving (RPS) form and the Discussion Tool, focused on identifying client-centered needs and clinical reasoning. In addition, two partners had electronic documentation systems that included the ICF but needed to be clarified and some aspects of the ICF perspective added (25).

## Discussion “education vs. training”

Our experience in developing the ICF training in the INPRO project clearly highlighted that formal ICF education provided by the HEIs is clearly not meeting the requirements of clinical practice. The importance of ICF training and materials in clinical setting is obvious, as clinical use is one of the main areas where the ICF has been used in different countries (10).

ICF training for the workplace was requested, with more practical examples and better integration into clinical practice. It was understandable that each rehabilitation center knew best what kind of training best suited its needs and resources. It can also be difficult for HEIs to to identify these needs. If HEIs consider the ICF as something to be taught without considering how it can be implemented in clinical practice or for other purposes, this is problematic, because formal ICF education provided in HEIs would consequently not meet the requirements of clinical practice. It is therefore important that there continues to be a lot of cooperation between higher education and clinical practice to better understand each other's needs. In this way, we can continue to bridge the gap between education and working life in the future.

It has been noted that the integration of the ICF into medical and healthcare education programs has been slow (17, 19), and its application still requires attention, training and support both in education and practice (14). We fully agree with these. In our experience, the ICF must be an integral part of the curriculum, and ICF terminology needs to be used from the day one of the education program. From the outset, students must learn to look at clients from a biopsychosocial approach and use ICF terminology in clinical reasoning and in their intra- and interprofessional communication. One aim of the ICF is to establish a common language between different users. The development of ICF training in the INPRO project identified two extreme groups of the professionals. First ones are newly graduated professionals who only “know” the ICF, but don't use the ICF after graduation. The other group comprise of professionals who graduated a long time ago and thus have a different ICF education or no ICF education at all. Finding a common language requires ICF training in clinical practice.

Ideally, and this is our dream for the future, ICF training should be available in every social and healthcare organization and easily transferable to clinical practice. Such training could use material based on real life clients, which could be further used in collaboration with HEIs and vice versa. HEIs could use the material developed in practice to teach students - future professionals. There would also be continuous lifelong learning in the workplace, according to the needs of each professional. The experience we have gained from developing the ICF training in the INPRO project shows that the more familiar professionals are with the ICF framework, the easier it may be to delve into specific topics that combine interprofessional collaboration and person-centeredness.

During the focus-groups, we noticed that the rehabilitation centers had quite similar needs and worked closely together on the project, but still each wanted to develop their own training and materials. This was surprising, as pooling resources could have been beneficial. This may be due to challenges of the project, such as limited human resources. Or perhaps the differences in rehabilitation practices between countries were so great that it was not possible to combine the materials. It seemed that the ICF framework was perceived to be used at slightly different learning levels in each country, which may also have contributed to this. However, we assume that training materials developed by one organization can be used in other organizations or countries. They can be used as an example for developing their own practices.

The INPRO project was based on two key ICF themes. First, evidence that the ICF framework can be used to define goals that cover all aspects of a person's life and can assist in clinical goal-setting processes (23). Secondly, it has been found that rehabilitation is not as person-centred as it should be (26). During the development of the materials and the training sessions in rehabilitation centers, professionals experienced a clear change in their practices. The facilitator of one rehabilitation center noted that the training was successful: “*Many colleagues nowadays spend more time and put more effort into formulating the main objective”*.

The professionals felt that the material developed helped them to look at the client's situation from a biopsychosocial perspective better than the previous ICF education. Participants recognized the value of being able to change their own perspective and approach as professionals. The training also opened up a reflection on what style of guidance would work best for the client in question and how person-centeredness is present. Feedback from rehabilitation professionals follows:

*“Implementing the ICF in work requires working on one*’*s own approaches, changing one*’*s own perspectives in the direction of the ICF.”*

*“Important: taking the whole client into account.”*

*“Consideration of the type of guidance style that is most beneficial to the client. What is this person made of?”*

In addition to providing rehabilitation professionals with tools to improve goal setting, one of the aims of the training material was to learn more about the ICF framework and its benefits for interprofessional collaboration. The ICF training also aimed to bring together interprofessional teams, to give professionals the opportunity to get to know each other and to learn how to use the common ICF language.

The interprofessional collaboration needs interaction with the client and her/his family, rehabilitation professionals and medical staff. It is important to facilitate the wider use of the ICF by medical professionals such as doctors and nurses. This could be easily done during the ICF training in clinical practice using training materials suitable for all professional groups. The development process of our INPRO project overcame many of the interprofessional barriers mentioned by Snyman et al. (27), such as over-reliance on learning modules from one profession, lack of common language and practices across professions, and limited opportunities for genuine interprofessional learning. The importance of creating a positive organizational culture for the adoption of ICF-based practices was identified.

Based on our experience from the INPRO project, we concluded that it is worth considering at what point it would be fruitful to provide ICF training in clinical practice. One important step in implementing the ICF in workplace is to train rehabilitation professionals in its use, this was done even though their knowledge of the ICF framework varied widely. The question is, would professionals get more out of ICF training if they had the same basic knowledge of the ICF framework? We believe that this is the case. It is thethe responsibility of the HEIs to provide their students with a thorough knowledge of the ICF as it is crucial for person-centred interprofessional practice. Our experience of the INPRO development process showed that HEI teachers' perceptions and experiences of the ICF varied a lot (24). The HEI teachers need be trained to incorporate the ICF and an integral approach in all their teaching.

We suggest that workplaces should also have highly educated ICF experts who could train other professionals in their organization. Peer support from professionals is also important. You don't need to know everything straight away. It is important to remember that the “I dońt know” can be a fruitful step towards learning something new from a different perspective. As an interprofessional workplace facilitator, it is also valuable to be prepared to deal with your own “I don't know” situations. The INPRO Process Guide for trainers in rehabilitation practice (28) draws attention to the fact that group discussion of uncertainties supports the development of a climate of trust. As Reed et al. (16) point out, the knowledge and skills of the ICF educator or facilitator in clinical practice are essential to resolving uncertainties.

## Conclusion

Based on our experience in developing ICF training in the INPRO project, we concluded that ICF training in clinical practice is needed to complement the formal ICF education offered in HEIs. The fact that the ICF education provided by HEIs does not meet the requirements of clinical practice may be due to shortcomings in the teaching of ICF to students (education) and specific requirements for teaching ICF to professionals already working in rehabilitation (training). In this paper we focused on the practical requirements in the workplace.

Workplaces requested ICF training with more practical examples and better integration into clinical practice. The innovation of the materials developed is that they focus on the application of the ICF in each organization's own clinical practice. The materials support ICF training by highlighting the holistic scope of the ICF and several options for developing client-oriented and interprofessional use of the ICF in rehabilitation centers.

Collaboration between higher education and clinical practice is important to better understand each other's practices and to bridge the gap between students' ICF education and the ICF training needed by clinical practice. Future developments could place even more emphasis on combining peer support and collaboration with mentoring of university supervisors. To deepen and broaden the integration of different ICF-based materials and practices, it is important to continue the interactive dialogue between management and employees.

We should remember the wise advice of Wade and Halligan (29):

*“The ICF also requires collaborative sharing and working across existing boundaries, which requires trust and sharing, an agreed understanding of the situation. This takes time to develop – years – and needs to be incremental. It also makes it obvious that patient-related factors are important.”*

## Data Availability

Publicly available datasets were analyzed in this study. This data can be found here: https://www.inproproject.eu/icf-based-tools-practices/.
